# Job involvement in heavy manual or physical work associated with epigenetic age: A Mendelian randomization study

**DOI:** 10.1097/MD.0000000000041541

**Published:** 2025-02-21

**Authors:** Xiqiao Sun

**Affiliations:** a Zhongshan School of Medicine, Sun Yat-sen University, Guangzhou, China.

**Keywords:** causal association, epigenetic age, genome-wide association study (GWAS), heavy manual or physical work, Mendelian randomization (MR)

## Abstract

Engaging in heavy manual or physical work has been linked to various health outcomes, but its effect on physiological aging, as measured by epigenetic clocks, remains unclear. This study aims to analyze the causal relationship between heavy manual or physical work and epigenetic age acceleration using Mendelian randomization (MR). In this study, we explored the causal link between job involvement in heavy manual or physical work and epigenetic age acceleration measured by 4 different epigenetic clocks using 2-sample MR. Our analytical approach included inverse-variance weighting (IVW), MR-Egger, weighted median, and weighted mode methods. The primary analyses utilized IVW with random effects, supplemented by sensitivity and heterogeneity tests using both IVW and MR-Egger. MR-Egger was also applied for pleiotropy testing. Additionally, a leave-one-out analysis helped identify potentially impactful single-nucleotide polymorphisms. The analysis revealed a positive association between heavy manual or physical work and epigenetic clock acceleration. There statistically significant associations between heavy manual or physical work with a higher PhenoAge and HannumAge acceleration (β = 1.692, 95% CI [0.349 to 3.035], *P* = .013 for PhenoAge; β = 0.917, 95% CI [0.024 to 1.809], *P* = .044 for HannumAge, respectively). The heterogeneity test revealed that our IVW analysis exhibited minimal heterogeneity (*P* > .05), and the pleiotropy test findings confirmed the absence of pleiotropy within our IVW analysis (*P* > .05). Our study provides partial evidence for a causal effect between heavy manual or physical work and epigenetic age acceleration. Further experimental research is required to confirm these findings.

## 1. Introduction

Heavy manual or physical work involves activities that require significant physical effort and exertion, which are associated with various health risks.^[1]^ Prolonged engagement in heavy manual labor can lead to retinal detachment,^[2]^ low back pain,^[3]^ cardiovascular risk^[4]^ and Alzheimer’s disease.^[5]^ Understanding the long-term health implications of such work, including its potential impact on biological aging processes, is essential for developing effective occupational health strategies.

Epigenetic clocks are biomarkers that use DNA methylation patterns to estimate biological age, offering insights into aging and related health outcomes.^[6]^ The first generation includes HannumAge,^[7]^ which assesses immune system aging using DNAm data from blood samples, and HorvathAge,^[8]^ a “pan-tissue” clock based on CpG sites across various tissues, providing a comprehensive measure of biological age. The second generation features PhenoAge,^[9]^ which combines DNAm data with clinical biomarkers to predict age-related diseases and mortality, and GrimAge,^[10]^ which incorporates DNAm data along with plasma proteins and smoking history to forecast lifespan and healthspan more accurately.

Work stress and physical activity level have important implications for longevity and diseases of aging,^[11]^ and previous observational studies have shown that low job control is associated with 1.40 years, heavy lifting with 2.08 years, and long working hours with 1.87 years of accelerated biological aging.^[12]^ However, the relationship between heavy manual or physical work and epigenetic age acceleration is not clear.

Accordingly, we designed a 2-sample Mendelian randomization (MR) study to investigate the relationship between heavy manual or physical work and epigenetic age acceleration. Mendelian randomization analysis leverages genetic variations as instrumental variables (IVs) to infer causal relationships between exposure factors and disease.^[13]^ By utilizing genome-wide association study (GWAS) data, MR can elucidate the association between specific factors and a range of health outcomes.^[14]^

## 2. Materials and methods

### 2.1. Study design

**Figure 1.**
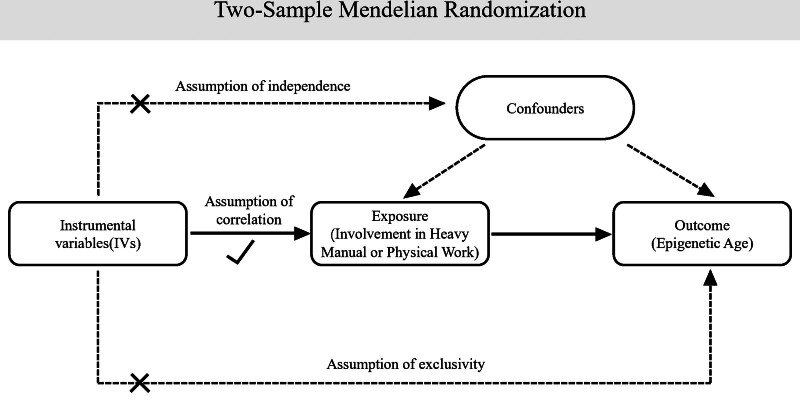
Assumptions and study design of the MR study of the associations between job involvement in heavy manual or physical work and epigenetic age. IVs = instrumental variables, MR = Mendelian randomization.

We conducted a 2-sample MR analysis to investigate the causal effect of heavy manual or physical work on epigenetic age acceleration. Genetic variants, serving as IVs, were selected from the summary statistics of large-scale GWAS. These IVs were utilized under the following assumptions: the IVs were significantly associated with heavy manual or physical work; the IVs were independent of confounding factors related to both heavy manual or physical work and epigenetic age; and the IVs influenced epigenetic age solely through their effect on heavy manual or physical work.^[13]^ Figure 1 shows an overview of the 3 hypotheses.

### 2.2. Data sources

We obtained summary genetic association estimates for epigenetic age acceleration measures from the recent GWAS biological aging meta-analysis: Intrinsic HorvathAge,^[8]^ HannumAge,^[7]^ PhenoAge,^[9]^ and GrimAge.^[10]^ The analysis was a meta-analysis based on the European ancestry of 34,710 participants from 28 cohorts, 57.3% of whom were women, and it identified 137 loci for DNA biomarkers associated with aging. For more information and a detailed description of the methodology, please see the latest GWAS meta-analysis of biological aging.^[15]^

The heavy manual or physical work GWAS summary statistics for European ancestry were obtained from the IEU OpenGWAS project (https://gwas.mrcieu.ac.uk). The database (ukb-a-503) contained 10,894,596 single-nucleotide polymorphisms (SNPs) with a sample size of 190,643 individuals. This study was a reevaluation of existing and publicly available data; therefore, no ethical authorization was necessary.

### 2.3. Genetic instrument variant (IV) selection

For the MR analysis, we selected suitable IVs from various GWAS summary data. We use “TwoSampleMR” package of R software to exclude the interference of chain imbalance, and the parameters were set at *P* < 5 × 10^−8^, *r*^2^ = 0.001, and kb = 10000. IVs with an *F* value < 10 were excluded from the analysis.^[16]^ To ensure the independence of the selected IVs, we used PhenoScanner (http://www.phenoscanner.medschl.cam.ac.uk/) to check for correlations between the SNPs and other phenotypes, such as diet and smoking.^[17]^

### 2.4. Statistical analysis

The inverse variance weighting (IVW) method employs meta-analysis techniques to combine the Wald estimates for each SNP,^[18]^ allowing us to evaluate the effect of heavy manual or physical work on epigenetic age acceleration. IVW was our primary statistical approach to examine the unidirectional relationship between heavy manual or physical work and epigenetic age. This method provides unbiased effect estimates when the selected IVs are valid, assuming no horizontal pleiotropy or heterogeneity is present. To complement the IVW results, we also applied the MR-Egger method,^[19]^ weighted median method (WME)^[20]^ and weighted mode.^[21]^

### 2.5. Sensitivity analysis

We used a variety of methods for sensitivity testing. Cochran’s *Q* test^[22]^ and MR egger intercept^[19]^ were used to test for heterogeneity and horizontal pleiotropy of results, and we conducted sensitivity analysis using the “leave-one-out” method, examining the impact of each SNP on the effect estimates by individually removing them one at a time.

The above statistical treatments were implemented by software R4.3.3 and TwoSampleMR package.

## 3. Results

After a strict selection process, 14 SNPs were chosen to conduct a 2-sample MR analysis of job involvement in heavy manual or physical work and epigenetic age acceleration (Table S1, Supplemental Digital Content, http://links.lww.com/MD/O415).

**Figure 2.**
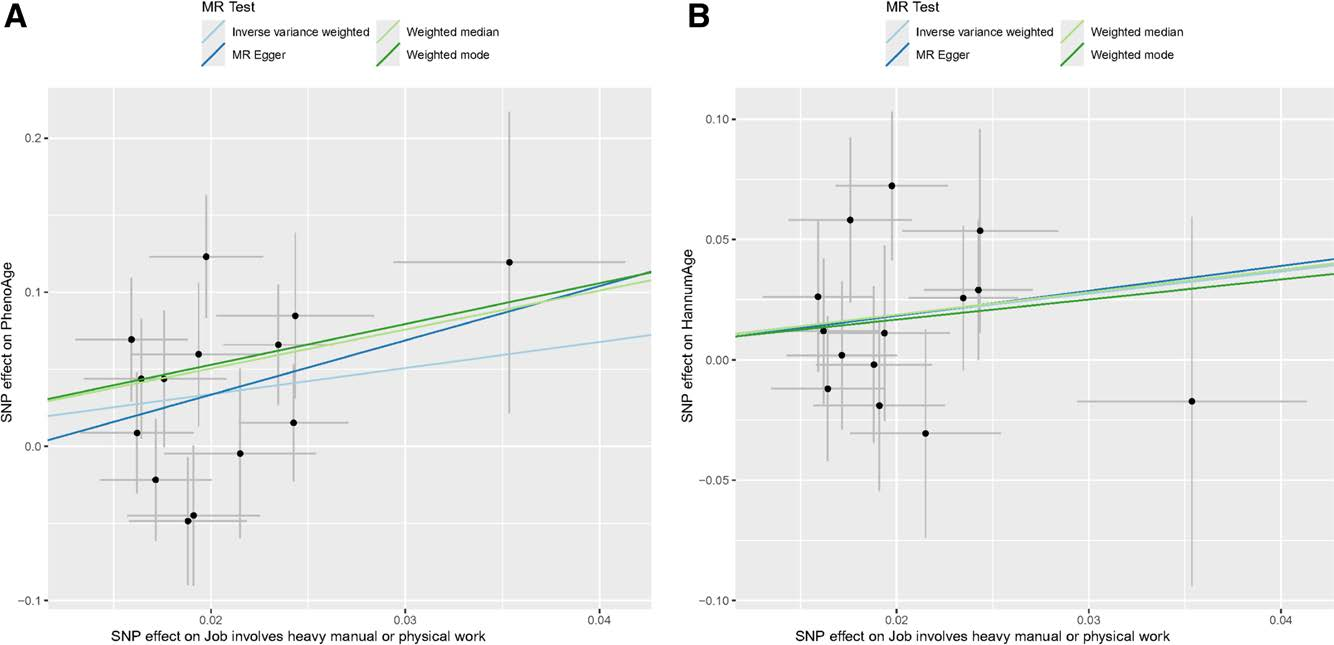
Scatter plots for 2 positive results in the 2-sample Mendelian randomization analyses. (A) Job involvement in heavy manual or physical work on PhenoAge. (B) Job involvement in heavy manual or physical work on HannumAge. MR = Mendelian randomization, SNP = single-nucleotide polymorphism.

**Figure 3.**
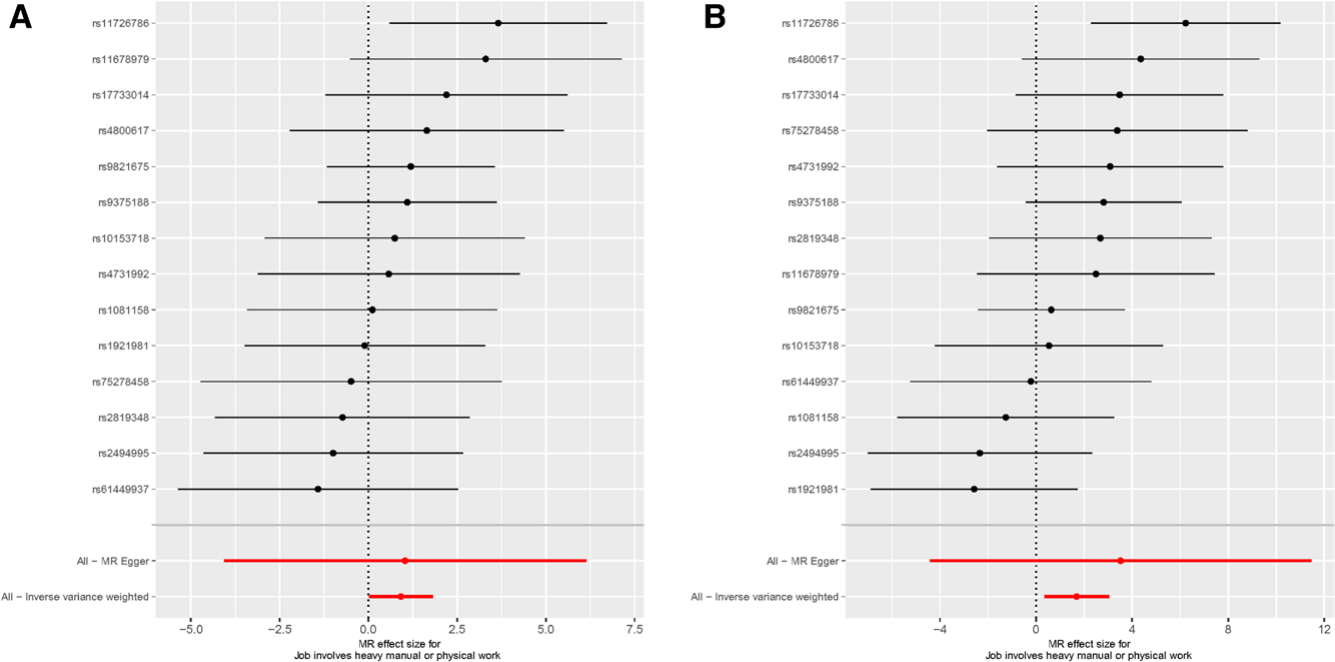
Forest plots for 2 positive results in the 2-sample Mendelian randomization analyses. (A) Job involvement in heavy manual or physical work on HannumAge. (B) Job involvement in heavy manual or physical work on PhenoAge.

The IVW method shows a positive correlation between job involvement in heavy manual or physical work and the epigenetic age acceleration of PhenoAge and HannumAge (Figs. 2 and 3; β = 1.692, 95% CI [0.349–3.035], *P* = .013 for PhenoAge; β = 0.917, 95% CI [0.024–1.809], *P* = .044 for HannumAge, respectively), with no statistically evidence of a relationship with HorvathAge and GrimAge (Table 1).

**Table 1:** Two-sample MR analyses results.

Exposure	Outcome	Method	NSNP	β	SE	*P*val
Job involvement in heavy manual or physical work	GrimAge	Inverse variance weighted	14	0.854 (−0.133~1.841)	0.503701	.089989
		MR Egger	14	3.725 (−1.943~9.394)	2.892421	.222009
		Weighted median	14	0.878 (−0.451~2.207)	0.678258	.19536
		Weighted mode	14	0.880 (−1.678~3.438)	1.305371	.512012
	HannumAge	Inverse variance weighted	14	0.917 (0.024~1.809)	0.455387	.044018
		MR Egger	14	1.034 (−4.071~6.139)	2.604873	.698262
		Weighted median	14	0.936 (−0.244~2.117)	0.602377	.120009
		Weighted mode	14	0.835 (−1.011~2.681)	0.942047	.391439
	HorvathAge	Inverse variance weighted	14	0.700 (−0.253~1.654)	0.486836	.150026
		MR Egger	14	4.850 (−0.418~10.120)	2.688564	.096334
		Weighted median	14	0.348 (−0.977~1.674)	0.676489	.606147
		Weighted mode	14	−0.142 (−2.520~2.235)	1.213154	.908163
	PhenoAge	Inverse variance weighted	14	1.692 (0.349~3.035)	0.685294	.013522
		MR Egger	14	3.520 (−4.413~11.454)	4.047934	.401499
		Weighted median	14	2.525 (0.886~4.163)	0.836114	.002527
		Weighted mode	14	2.644 (−0.322~5.610)	1.513672	.104242

MR = Mendelian randomization, NSNP = number of single-nucleotide polymorphisms, SE = standard error.

**Figure 4.**
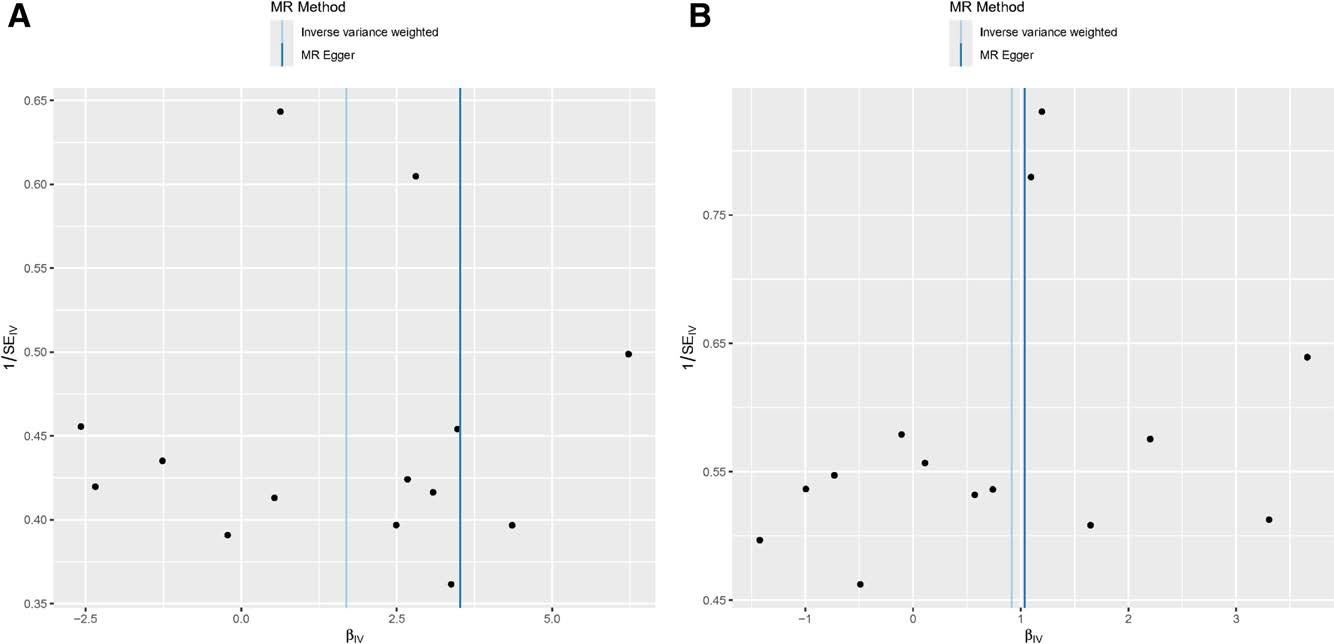
Funnel plots for 2 positive results in the 2-sample Mendelian randomization analyses. (A) Job involvement in heavy manual or physical work on PhenoAge. (B) Job involvement in heavy manual or physical work on HannumAge.

**Figure 5.**
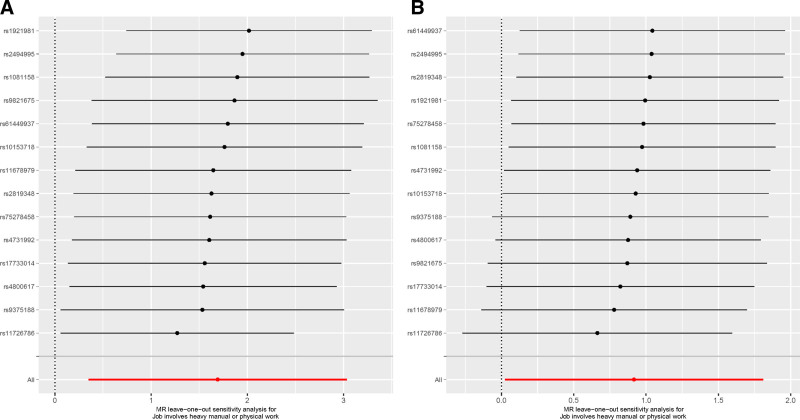
Leave-one-out analysis. (A) Job involvement in heavy manual or physical work on PhenoAge. (B) Job involvement in heavy manual or physical work on HannumAge.

In sensitivity analysis, Cochran’s *Q* test indicates no heterogeneity in the results. Furthermore, the MR Egger intercept results suggest no horizontal pleiotropy Figure 4. The leave-one-out method did not reveal any SNP significantly biasing the overall MR results (Fig. 5).

## 4. Discussion

Our study explored the causal relationship between job involvement in heavy manual or physical work and epigenetic age acceleration using a 2-sample MR approach. The results indicated a positive association between heavy manual or physical work and accelerated epigenetic aging, specifically with PhenoAge and HannumAge clocks, suggesting that individuals engaged in such work may experience accelerated biological aging.

The observed associations align with previous findings that link physically demanding work with adverse health outcomes, including osteoarthritis^[23]^ and cardiovascular diseases.^[4]^ This study extends these findings by providing evidence that heavy manual or physical work may also contribute to accelerated epigenetic aging, an important marker of overall biological aging and age-related diseases.^[24]^ The lack of association with HorvathAge and GrimAge suggests that the impact of heavy manual or physical work on biological aging may be more pronounced in specific epigenetic clocks, which could reflect different aspects of aging biology.^[25]^

Several mechanisms could explain the observed association. Heavy manual labor is often accompanied by chronic physical stress^[26]^ and inflammation,^[27]^ which are known to influence epigenetic modifications and accelerate aging processes.^[24]^ Additionally, such work may be associated with lifestyle factors, such as poor diet, inadequate sleep, and higher exposure to environmental toxins, which can further contribute to accelerated epigenetic aging.^[24]^

The robustness of our findings is supported by sensitivity analyses. The absence of heterogeneity and horizontal pleiotropy, as indicated by Cochran’s *Q* test and MR-Egger intercepts, reinforces the validity of our causal estimates. Moreover, the leave-one-out analysis confirmed that no single SNP disproportionately influenced the results, ensuring the reliability of our conclusions.

However, this study has limitations. First, the use of GWAS summary statistics limits the ability to explore potential interactions between genetic variants and environmental factors in detail. Second, the analysis is based on data from individuals of European ancestry, which may limit the generalizability of our findings to other populations. Future research should aim to replicate these findings in diverse populations and further investigate the underlying biological mechanisms.

## 5. Conclusions

In conclusion, our study provides evidence for a positive causal relationship between heavy manual or physical work and epigenetic age acceleration, particularly as measured by PhenoAge and HannumAge. These findings suggest that individuals engaged in physically demanding work may be at risk for accelerated biological aging, highlighting the importance of occupational health interventions to mitigate these effects. Further experimental and longitudinal studies are needed to confirm these results and to explore the potential pathways through which heavy manual labor impacts epigenetic aging.

By addressing these questions, we can better understand the broader implications of work-related stressors on aging and develop strategies to promote healthier aging in the workforce.

## Acknowledgments

We are grateful to the IEU OpenGWAS databases for providing the data platform, to the authors of the data, and to the developers of the R software and related packages used in the article for MR analysis.

## Author contributions

**Conceptualization:** Xiqiao Sun.

**Data curation:** Xiqiao Sun.

**Formal analysis:** Xiqiao Sun.

**Funding acquisition:** Xiqiao Sun.

**Investigation:** Xiqiao Sun.

**Methodology:** Xiqiao Sun.

**Project administration:** Xiqiao Sun.

**Resources:** Xiqiao Sun.

**Software:** Xiqiao Sun.

**Supervision:** Xiqiao Sun.

**Validation:** Xiqiao Sun.

**Visualization:** Xiqiao Sun.

**Writing – original draft:** Xiqiao Sun.

**Writing – review & editing:** Xiqiao Sun.

## Supplementary Material


